# Supplementary material for: End Sequence Analysis Toolkit (ESAT) expands the extractable information from single-cell RNA-seq data
Source: Genome Res. 2016 Oct;26(10):1397–410. doi: 10.1101/gr.207902.116 (PMC5052061; doi:10.1101/gr.207902.116)
Supplement: Supplemental Material [file supp_gr.207902.116_Supp_Materials.tar.gz › ESAT-esat_v0.1/doc/html/classbroad_1_1core_1_1parser_1_1_command_line_parser-members.html]

ESAT: Member List


ESAT


- Main Page
- Related Pages
- Packages
- Classes
- Files

- Class List
- Class Index
- Class Hierarchy
- Class Members

All Classes Namespaces Files Functions Variables Enumerator Pages

- broad
- core
- parser
- CommandLineParser

broad.core.parser.CommandLineParser Member List

This is the complete list of members for broad.core.parser.CommandLineParser, including all inherited members.

|  |  |  |
| --- | --- | --- |
| addBooleanArg(String flag, String description, boolean required) | broad.core.parser.CommandLineParser |  |
| addBooleanArg(String flag, String description, boolean required, boolean def) | broad.core.parser.CommandLineParser |  |
| addDoubleArg(String flag, String description, boolean required) | broad.core.parser.CommandLineParser |  |
| addDoubleArg(String flag, String description, boolean required, double def) | broad.core.parser.CommandLineParser |  |
| addFloatArg(String flag, String description, boolean required) | broad.core.parser.CommandLineParser |  |
| addFloatArg(String flag, String description, boolean required, float def) | broad.core.parser.CommandLineParser |  |
| addIntArg(String flag, String description, boolean required) | broad.core.parser.CommandLineParser |  |
| addIntArg(String flag, String description, boolean required, int def) | broad.core.parser.CommandLineParser |  |
| addStringArg(String flag, String description, boolean required) | broad.core.parser.CommandLineParser |  |
| addStringArg(String flag, String description, boolean required, String def) | broad.core.parser.CommandLineParser |  |
| addStringListArg(String flag, String description, boolean required, ArrayList< String > def) | broad.core.parser.CommandLineParser |  |
| addStringListArg(String flag, String description, boolean required) | broad.core.parser.CommandLineParser |  |
| CommandLineParser() | broad.core.parser.CommandLineParser |  |
| getArgString() | broad.core.parser.CommandLineParser |  |
| getArgString(Collection< String > flagsToRemove) | broad.core.parser.CommandLineParser |  |
| getBooleanArg(String flag) | broad.core.parser.CommandLineParser |  |
| getDoubleArg(String flag) | broad.core.parser.CommandLineParser |  |
| getFlagsAndValues() | broad.core.parser.CommandLineParser |  |
| getFloatArg(String flag) | broad.core.parser.CommandLineParser |  |
| getIntArg(String flag) | broad.core.parser.CommandLineParser |  |
| getStringArg(String flag) | broad.core.parser.CommandLineParser |  |
| getStringListArg(String flag) | broad.core.parser.CommandLineParser |  |
| hasBooleanFlag(String flag) | broad.core.parser.CommandLineParser |  |
| hasDoubleFlag(String flag) | broad.core.parser.CommandLineParser |  |
| hasFloatFlag(String flag) | broad.core.parser.CommandLineParser |  |
| hasIntFlag(String flag) | broad.core.parser.CommandLineParser |  |
| hasStringFlag(String flag) | broad.core.parser.CommandLineParser |  |
| parse(String[] args, boolean allowDuplicateTags) | broad.core.parser.CommandLineParser |  |
| parse(String[] args) | broad.core.parser.CommandLineParser |  |
| printHelpMessage() | broad.core.parser.CommandLineParser |  |
| setProgramDescription(String description) | broad.core.parser.CommandLineParser |  |


---

Generated on Fri Aug 15 2014 15:10:55 for ESAT by  

 1.8.7
